# Assisted reproductive technologies in Latin America: the Latin American Registry, 2019

**DOI:** 10.5935/1518-0557.20220034

**Published:** 2022

**Authors:** Fernando Zegers-Hochschild, Javier A. Crosby, Carolina Musri, Maria do Carmo Borges de Souza, A. Gustavo Martinez, Adelino Amaral Silva, José María Mojarra, Diego Masoli, Natalia Posada, Latin American Network of Assisted Reproduction

**Affiliations:** 1 Unit of Reproductive Medicine, Clínica Las Condes, Santiago, Chile; 2 Program of Ethics and Public Policies in Human Reproduction, Facultad de Medicina, Universidad Diego Portales, Santiago, Chile; 3 Fertipraxis, Barra da Tijuca Rio de Janeiro, Brazil; 4 Fertilis, San Isidro Provincia de Buenos Aires, Argentina; 5 Genesis-Centro de Assistência em Reprodução Humana, SHLS cj L, Brasília, Brazil; 6 Hospital CIMA Hermosillo, Col. Proyecto Rio Sonora C.P., Hermosillo Sonora 83280, Mexico; 7 INSER, Sector El Poblado Medellín, Colombia; 8 Latin American Network of Assisted Reproduction (REDLARA) Montevideo, Uruguay

**Keywords:** ART utilization, assisted reproductive technology, efficacy Latin American registry, perinatal outcome

## Abstract

**Research question:**

What was the utilization, effectiveness and safety of assisted reproductive technology (ART) in Latin America during 2019?

**Design:**

This was a retrospective collection of multinational data on ART performed at 196 institutions from 15 countries.

**Results:**

A total of 106,918 initiated cycles, 18,133 deliveries and 21,096 births were reported. ART utilization was 24-558 cycles per million inhabitants. Women aged ≥40 years represented 32.9% of fresh IVF and intracytoplasmic sperm injection (ICSI) cycles. After removing freeze-all cycles, the delivery rate per oocyte retrieval was 17.3% for ICSI and 19.5% for IVF. Overall, single-embryo transfer (SET) represented 36.2% of fresh transfers, with a 19.5% delivery rate per transfer, increasing to 30.7% for elective SET and 32.7% for blastocyst elective SET (eSET). The delivery rate for double-embryo transfers (DET) was 27.8%, increasing to 37.1% after elective DET. This 6.4% increment in deliveries between eSET and elective DET resulted in a 12-fold increase in twin births. Furthermore, overall perinatal mortality was more than two-fold higher for twin compared with singleton deliveries. The delivery rate for frozen-thawed SET reached 28.1%, most being blastocyst transfers. Of all births, 72.3% were singletons, 26.4% twins and 1.3% triplets and higher multiples. Preterm deliveries reached 14.3% for singletons and 58.1% for twins. Perinatal mortality was 7.4‰ in singletons, 17.2‰ for twins and 62.9‰ for triplets or higher.

**Conclusions:**

The number of initiated cycles has slowly increased in countries with laws or regulations facilitating access. FET cycles predominate and blastocyst SET are also increasing. The data show that, especially in young women and oocyte recipients, when there is more than one blastocyst for transfer, eSET should be the rule.

## INTRODUCTION

This is the 31^st^ report of the Latin American Registry of Assisted Reproduction (RLA), established in 1990 as the first multinational and regional registry of assisted reproductive technology (ART). Since 2012, reports have been published simultaneously in *Reproductive BioMedicine Online RBMO* and *JBRA Assisted Reproduction*, the official journal of the Latin American Network of Assisted Reproduction (REDLARA). The results from previous years can be downloaded from www.redlara.com. This report provides information on the utilization, availability, effectiveness, safety and perinatal outcomes of ART treatments initiated between 1 January and 31 December 2019, and babies born up to September 2020.

## MATERIALS AND METHODS

Data on ART were collected from 196 centres in 15 countries in Latin America (Supplementary Table 1), covering: fresh autologous cycles involving IVF and intracytoplasmic sperm injection (ICSI); preimplantation genetic testing (PGT); frozen embryo transfer (FET) preceded by either fresh embryo transfer cycles or freeze-all cycles; oocyte donation, including the transfer of fresh and frozen-thawed embryos; fertility preservation; and vitrified-warmed oocyte (FTO) cycles, both autologous and heterologous. This report includes treatments started between 1 January 2019 and 31 December 2019. Data on pregnancy and perinatal outcomes were obtained from a follow-up of cohorts treated during this period.

As part of the accreditation programme, all participating institutions agree to have their data registered and published by the RLA. Therefore, no other consent form was requested for the scientific disclosure of these data.

The method of data collection in 2019 resembled that of previous years (Zegers-Hochschild *et al*., 2020), making the results comparable. The definitions used refer to the latest publication of the International Glossary on Infertility and Fertility Care (Zegers-Hochschild *et al*., 2017). When calculating CPR or delivery rate per oocyte retrieval, cases resulting in total embryo freezing were not included in the calculation.

The cumulative live birth rate was calculated, as described by Maheshwari and colleagues (Maheshwari *et al*., 2015), from cycles taking place between 2017 and 2019 and considering the first delivery after the transfer of either fresh or frozen-thawed embryos, or both, obtained after a reference oocyte retrieval. A personal identification number and date of birth identified each woman. As the use of a fixed identification number is not universal in Latin America, not all women could be followed, and it is also possible that cross-border reproductive treatments could partially influence the results, but those numbers should be small. Furthermore, only data provided by institutions using a consistent and reproducible identification number were included throughout the study period (2017-2019). For the purpose of reporting cumulative births, 136 institutions in 14 countries were included (Nicaragua being excluded).

To test for the effect of age, the number of embryos transferred and the state of embryo development at transfer on the delivery rate per embryo transfer (DR/ET), logistic regression analysis was conducted for fresh and oocyte donation cycles. When appropriate, a Chi-squared test was used to analyse the independence of categorical variables. A value *p*<0.05 was considered statistically significant.

## RESULTS

### Participation

A total of 196 centres in 15 countries reported ART procedures carried out during 2019. This represents more than 85% of cycles in the region. Most centres were located in Brazil (*n*=63), followed by Mexico (*n*=40) and Argentina (*n*=28; Table 1). Compared with 2018, five centres that had stopped reporting resumed their participation, and six centres were newly incorporated in 2019, contributing more than 1100 of the 2749 new cycles reported in this period.

**Table 1:** Assisted reproduction techniques reported in Latin America, 2019.

Country	Centers	FP	Fresh	FET	OD	FTO	Total	Births registered by RLA	Estimated total number of births from ART	Estimated proportion of births from ART/total births in the country
Argentina	28	992	9714	3888	6333	482	21,409	3458	3562	0.57
Bolivia	3	5	355	32	273	25	690	152	236	0.10
Brazil	63	4048	21,663	15,598	3525	1911	46,745	8216	8545	0.30
Chile	11	603	2272	1382	821	347	5425	1204	1481	0.70
Colombia	15	162	1477	819	755	124	3337	982	1292	0.20
Ecuador	7	103	565	298	346	46	1358	331	407	0.14
Guatemala	2	29	157	108	123	14	431	107	141	0.04
Mexico	40	628	6769	3599	4548	340	15,884	4316	5352	0.26
Nicaragua	1	16	69	29	17	9	140	18	21	0.01
Panama	4	77	547	326	189	24	1163	278	375	0.52
Paraguay	1	97	131	118	55	17	418	63	84	0.11
Peru	13	1158	2766	1458	1863	824	8069	1606	1686	0.29
Dominican Republic	2	4	90	42	82	5	223	82	93	0.08
Uruguay	2	65	580	435	296	56	1432	358	451	1.20
Venezuela	4	3	86	52	51	2	194	68	109	0.02
Total (%)	196	7990 (7.5)	47,241 (44.2)	28,184 (26.4)	19,277 (18.0)	4226 (4.0)	106,918	-	-	-

ART, assisted reproductive technology; FET, autologous frozen embryo transfer; FP, fertility preservation; Fresh, initiated fresh autologous IVF and intracytoplasmic sperm injection cycles; FTO, embryo transfer cycles with autologous and donated vitrified/warmed oocytes; OD, oocyte donation with fresh or frozen/thawed embryos; RLA, Latin American Registry of Assisted Reproduction.

### Size of participating institutions and number of treatment cycles per technique

A total of 106,918 initiated cycles were reported during 2019 (2.6% more than in 2018). The mean number of initiated cycles by institution was 545.5, with a wide variation: 11.7% carried out ≤100 cycles; 30.6% between 101 and 300 cycles; 20.9% between 301 and 500 cycles; 18.4% between 501 and 1000 cycles; and 18.4% >1000 cycles. Overall, the major contributors were Brazil followed by Mexico and Argentina.

Out of 106,918 initiated cycles, 47,241 corresponded to IVF/ICSI (44.2%), 28,184 corresponded to FET (26.4%), 19,277 to oocyte donation (18.0%), 7990 to fertility preservation (7.5%) and 4226 to FTO (4.0%; Table 1).

**Figure 1:**
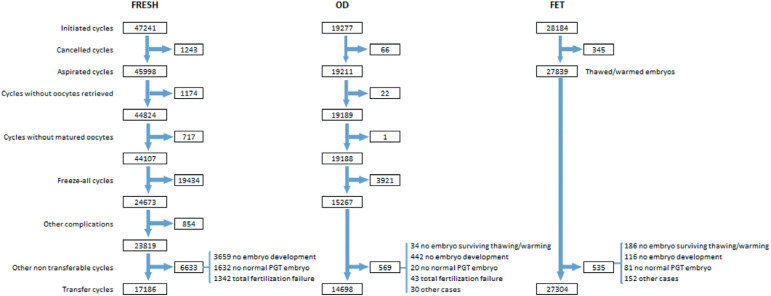
Events that affect the outcome of fresh IVF and intracytoplasmic sperm injection, oocyte donation (OD) with fresh and frozen-thawed embryo transfer, and autologous frozen embryo transfer (FET) in Latin America, 2019. PGT, preimplantation genetic testing.

A detailed description of the sequence of events that need to be considered when looking at the outcome of any specific technique (IVF/ICSI, oocyte donation, FET) is presented in Figure 1, starting with the initiated cycle; then cancellations before follicle aspiration; aspirations with or without mature oocytes; freeze-all oocytes, embryos or both; the number of cycles with fertilized oocytes or failed fertilization; and the number of cycles with viable embryos for transfer or normal embryos after PGT. It is only after all these events have been considered and adjusted that pregnancy and delivery rates can be calculated with a well-established denominator, this being the initiated cycle, aspirated cycle and transfer cycle. This detailed description is, however, only possible in a cycle-based registry.

### Utilization of ART in Latin America

The utilization of ART is expressed as the total number of cycles performed per million inhabitants. Considering that not all cycles carried out in every country were reported to the Latin American registry, the best possible estimate of the non-reported cycles was obtained through information provided by the regional directors of REDLARA, biologists, clinicians and industry representatives. The magnitude of the estimates, which constitutes a potential source of error, was expressed as degrees of confidence according to Dyer and colleagues (Dyer *et al*., 2019) and later applied by Zegers-Hochschild and co-workers (Zegers-Hochschild *et al*., 2021).

**Figure 2:**
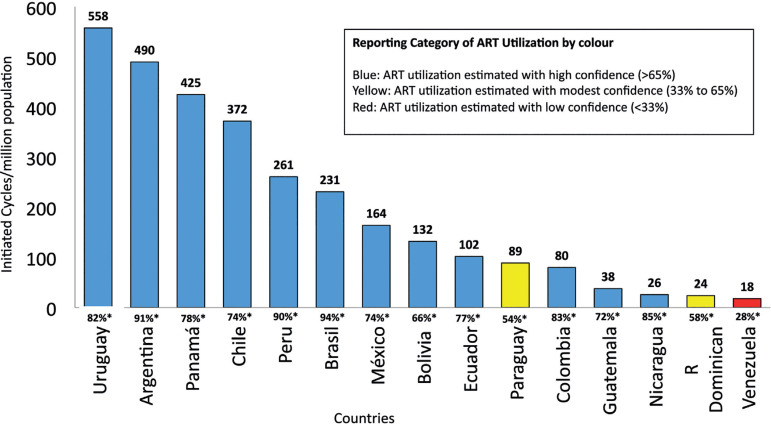
Utilization of assisted reproductive technology (ART). Estimated number of initiated cycles per million inhabitants by country in Latin America, 2019.

As seen in Figure 2, the RLA collects between 72% and 94% of ART cycles carried out in most countries in the region, and in particular the major contributors in Latin America are within this range. Overall, Argentina and Uruguay, two countries with laws providing universal ART care, have the highest utilization, with 490 and 558 cycles per million inhabitants respectively. Brazil is by far the major contributor in the region, but its utilization is still very poor.

### Age of women treated in Latin America

**Figure 3:**
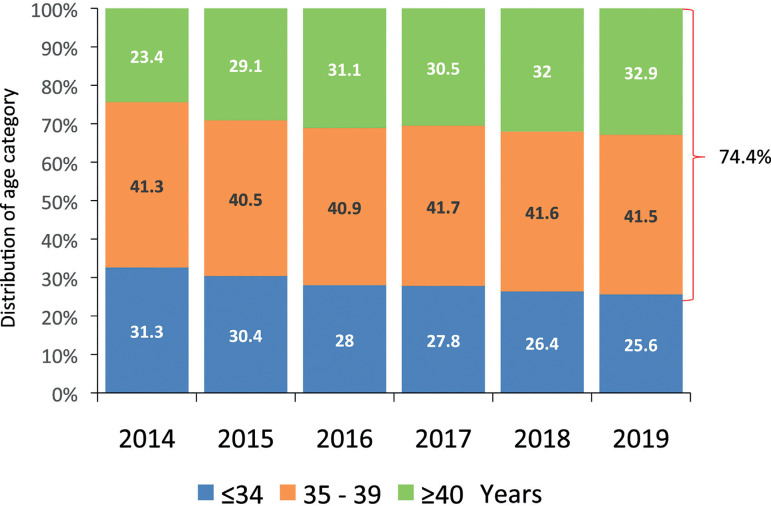
Age distribution of the female partner in fresh IVF and intracytoplasmic sperm injection (IVF/ICSI) in Latin America, 2014-2019.

In autologous reproduction the mean age of women undergoing IVF/ ICSI was 37.2 years (SD 4.49) years. Most cycles were carried out in women aged between 35 and 39 years (41.5%), followed by women aged ≥40 years (32.9%). Therefore, 74.4% of women using autologous ART were ≥35 years of age. Trends over the past 30 years are described by Zegers-Hochschild and co-workers (Zegers-Hochschild *et al*., 2021). However, in the past 6 years the trend of an ageing population has been seen. As seen in Figure 3, there has been a steady fall in the proportion of women aged ≤34 years, reaching only 25.6%, while the percentage women aged ≥40 years increased from 23.4% in 2014 to 32.9% in 2019. Furthermore, in oocyte recipients, the mean age of women was 42.1 (SD 4.88) years, and most cycles (59.2%) were carried out in women aged ≥42 years.

### Outcome of autologous fresh IVF and ICSI cycles

In 2019, 47,241 IVF/ICSI cycles were initiated. After discarding aspirations without oocytes or with an absence of mature oocytes, and 19,434 cycles of total embryo/oocyte freezing (Figure 1), 24,673 oocyte retrievals were exposed to the chance of pregnancy. There were 17,186 embryo transfer cycles generating 5770 clinical pregnancies (clinical pregnancy rate [CPR] 23.4% per oocyte retrieval and 33.6% per embryo transfer). Of these pregnancies, 64 were ectopic (1.11%), six were induced abortions (0.1%) and 1038 were miscarriages (18.0%). A total of 468 pregnancies were lost to follow-up (8.1%) and 4194 deliveries were reported.

The CPR and delivery rate per oocyte retrieval and embryo transfer in IVF and ICSI cycles are presented in Table 2. Of all the fresh procedures, ICSI represents 84.6%, and significant differences were reported in the CPR and delivery rate per oocyte retrieval between ICSI and IVF (23.7% and 27.1%, *p*<0.0001, and 17.3% and 19.5%, *p*=0.0014, respectively). However, when calculated in relation to embryo transfer (2971 in IVF and 14,215 in ICSI), the DR/ET in IVF (24.0%) and ICSI (24.5%) did not show a significant difference.

**Table 2:** Clinical pregnancy rate and delivery rate in fresh autologous IVF and intracytoplasmic sperm injection (IVF/ICSI) cycles in 2019.

Assisted reproduction technique procedure	Oocyte retrievalsa	Clinical pregnancy rate per oocyte retrieval	Delivery rate per oocyte retrieval	Embryo transfers	Delivery rate per embryo transfer
ICSI, n (%)	20,153	4777 (23.7)	3480 (17.3)	14,215	3480 (24.5)
IVF, n (%)	3666	993 (27.1)	714 (19.5)	2971	714 (24.0)
Total, n (%)	23,819	5770 (24.2)	4194 (17.6)	17,186^c^	4194 (24.4)
*P*-value (95% CI)^b^	-	<0.0001 (1.85-4.98)	0.0014 (0.82-3.62)	-	0.5799 (-1.23 to 2.19)

a Oocyte retrieval with at least one mature oocyte, excluding other complications and freeze-all cycles.

b IVF versus ICSI.

c This includes 9199 cleaving-stage embryos, 7963 blastocysts and 24 zygotes.

ICSI, intracytoplasmic sperm injection.

The overall numbers of embryos transferred and multiple births after IVF/ ICSI are presented in Table 3. The mean number of embryos transferred was 1.75 (range 1-6). There were 6225 single-embryo transfers (SET; 36.2%), 9250 double-embryo transfers (DET; 53.8%) and 1711 transfers with three or more embryos (9.96%).

**Table 3:** CPR, delivery rate and gestational order according to the number of embryos transferred in fresh autologous ICF and intracytoplasmic sperm injection cycles in 2019.

Number of transferred embryos	Embryo transfers		Clinical pregnancies		Deliveries							
	Number	%	Number	(%)	Number of deliveries	Delivery rate per embryo transfer (%)	Singleton (n)	Singleton (%)	Twin (n)	Twin (%)	≥Triplet (n)	≥Triplet (%)
1	6225	36.2	1670	26.8	1213	19.5	1187	97.9	26	2.1	0	0
2	9250	53.8	3486	37.7	2568	27.8	2011	78.3	551	21.5	6	0.2
≥3	1711	10.0	614	35.9	413	24.1	305	73.8	89	21.5	19	4.6
Total	17,186	100.0	5770	33.6	4194	24.4	3503	83.5	666	15.9	25	0.6

CPR, clinical pregnancy rate.

### Elective over non-elective embryo transfer in fresh autologous cycles

Overall, the delivery per embryo transfer reached 24.4%. In terms of multiple births, of the 4194 IVF/ICSI deliveries registered, 83.5% were singletons, 15.9% were twins and 0.6% were triplets or more (Table 3).

Given that SET constitutes a heterogeneous group, the outcomes of IVF and ICSI were further stratified after transfer related to eSET compared with oSET (the transfer of only one embryo because there are no more embryos available for transfer) and eDET compared with oDET (the transfer of only two embryos because there are no more embryos available for transfer; Table 4). Huge differences were found in the DR/ET for both eSET and eDET over oSET and oDET; furthermore, the rate of twins and triplets increased with eDET, whereas eSET by itself did not seem to increase the rate of monozygotic twins. As expected, these differences were even greater in the subset of women in whom only blastocysts were transferred. As seen in Table 5 eSET of blastocysts reached a CPR rate of 42.3% and a delivery rate of 32.7%.

**Table 4:** CPR, delivery rate and gestational order in elective and non-elective SET and DET in fresh autologous IVF/ICSI in 2019.

Transfer type	Embryo transfers		Clinical pregnancies		Deliveries							
	Number	%	Number	CPR	Number of deliveries	Delivery rate per embryo transfer (%)	Singleton (n)	Singleton (%)	Twin (n)	Twin (%)	≥Triplet (n)	≥Triplet (%)
oSET	3958	63.6	754	19.1	517	13.1	505	97.7	12	2.3	0	0
eSET	2268	36.4	916	40.4	696	30.7	682	98.0	14	2.0	0	0
oDET	5524	59.7	1679	30.4	1185	21.5	978	82.5	207	17.5	0	0
eDET	3725	40.3	1807	48.5	1383	37.1	1033	74.7	344	24.9	6	0.4

CPR, clinical pregnancy rate; eDET, elective double-embryo transfer; eSET, elective single-embryo transfer; ICSI, intracytoplasmic sperm injection; oDET, transfer of only two embryos because there are no more embryos available for transfer; oSET, transfer of only one embryo because there are no more embryos available for transfer.

**Table 5:** CPR, delivery rate and gestational order in elective and non-elective blastocyst SET and blastocyst DET in fresh autologous IVF/ICSI in 2019.

Transfer type	Embryo transfers		Clinical pregnancies		Deliveries							
	Number	%	Number	%	Number of deliveries	Delivery rate per embryo transfer (%)	Singleton (n)	Singleton (%)	Twin (n)	Twin (%)	≥Triplets (n)	≥Triplets (%)
oSET	1616	46.5	415	25.7	288	17.8	279	96.9	9	3.1	0	0
eSET	1858	53.5	786	42.3	607	32.7	596	98.2	11	1.8	0	0
oDET	2025	49.4	744	36.7	511	25.2	402	78.7	109	21.3	0	0
eDET	2072	50.6	1093	52.8	845	40.8	609	72.1	233	27.6	3	0.4

CPR, clinical pregnancy rate; eDET, elective double-embryo transfers; eSET, elective single-embryo transfers; ICSI, intracytoplasmic sperm injection; oDET, the transfer of only two embryos because there are no more embryos available for transfer; oSET, transfer of only one embryo because there are no more embryos available for transfer.

**Figure 4:**
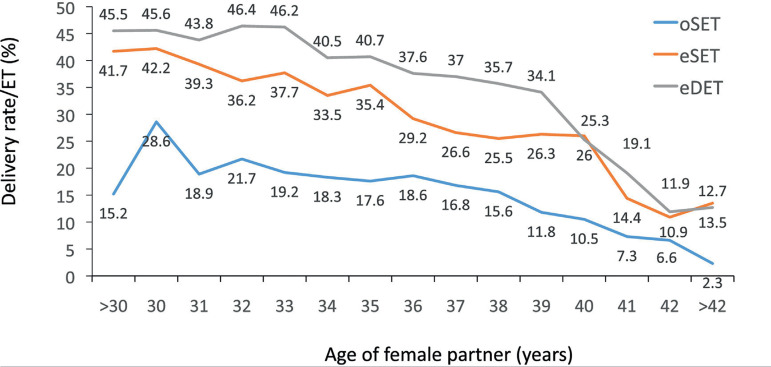
Delivery rate per embryo transfer (ET) in IVF and intracytoplasmic sperm injectioncycles according to the age of the female partner and the number of embryos transferred in Latin America, 2019. eDET, elective double-embryo transfer; eSET, elective double-embryo transfer; oDET, transfer of only two embryos because there were no more embryos available for transfer.

Furthermore, when the delivery rate was stratified according to the woman’s age, after transfer in oSET, eSET and eDET, women with the capacity to generate multiple embryos had a higher chance of birth than women generating only one embryo. This becomes evident at every age category when comparing eSET with oSET. Furthermore, except for women aged ≥40 years, the DR/ET increased when transferring two embryos over one (Figure 4).

### Outcome of oocyte donation cycles

As seen in Figure 1, in 2019, 19,277 cycles were initiated, and, after removing freeze-all cycles (oocytes and embryos) and those without suitable embryos for transfer, there were 14,698 transfer cycles. As expected, both the CPR and the delivery rate per embryo transfer were much higher after the transfer of donated oocytes (Table 6) than in autologous reproduction (Table 2), reaching 47.0% and 34.9%, respectively (CPR: *p*<0.0001; 95% confidence interval [CI] 11.93-14.67%; delivery rate: *p*<0.0001; 95% CI 9.21-11.8%). When compared with autologous reproduction in a selected group of women aged <35 years, the DR/ET (1617/4737, 34.1%) was not significantly different from that of oocyte recipients (*p*=0.3230; 95% CI -0.77% to 2.36%). Furthermore, when considering all oocyte donation cycles DR/ET was significantly higher after fresh transfers than after FET transfers (34.9% and 32.1%, respectively; *p*=0.0004; 95% CI 1.25-4.35%; Table 6).

**Table 6:** Clinical pregnancy rate and delivery rate by embryo transfer in oocyte donation and frozen embryo transfer cycles in 2019.

Assisted reproductive technology procedure	Embryo transfers (n)	Clinical pregnancy per embryo transfer (n, %)	Delivery rate per embryo transfer (n, %)
Fresh oocyte donation	6295	2957 (47.0)	2194 (34.9)
Frozen-thawed embryo transfer (oocyte donation)	8403	3591 (42.7)	2694 (32.1)
Frozen-thawed embryo transfer (own)	27,304	11,100 (40.7)	8196 (30.0)

**Figure 5:**
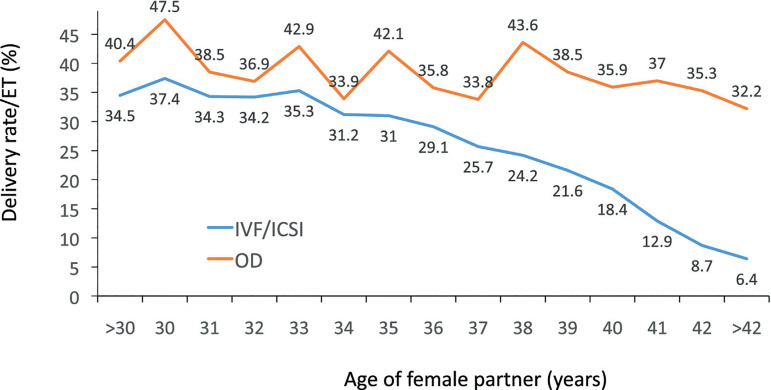
Delivery rate per embryo transfer (ET) in fresh autologous IVF and intracytoplasmic sperm injection (ICSI) and fresh oocyte donation (OD) cycles according to the age of the female partner in Latin America, 2019.

CPR, delivery rate and rate of multiple births according to the numbers of embryos transferred in both fresh oocyte donation (6295 transfers) and FET oocyte donation (8403 transfers) can be seen in Supplementary Tables 2 and 3. Multiple births were also higher after fresh oocyte donation (22.2%) than FET oocyte donation (16.2%). Furthermore, compared with autologous transfers, the chances of becoming pregnant and delivering after the use of oocyte donation is only marginally affected by the age of the oocyte recipient (Figure 5).

### Outcome of FET cycles

**Figure 6:**
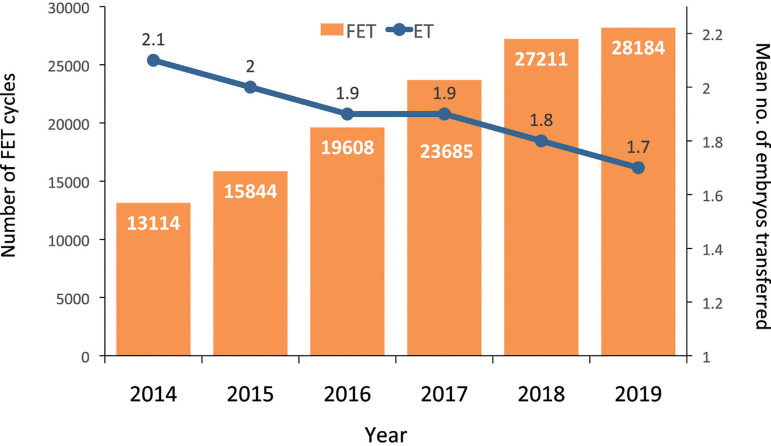
Number of frozen embryo transfer (FET) cycles and mean number of embryos per transfer (ET) in Latin America, 2014-2019.

In 2019, there were 28,184 FET cycles, representing 26.4% of all procedures. This represents an increment of more than 114% compared with 2014. In this same time interval, the overall mean number of embryos transferred dropped from 2.1 in 2014 to 1.7 (Figure 6).

Of all the initiated FET cycles, 880 (3.1%) cycles were discontinued. Reasons for discontinuation included embryos not surviving after thawing/warming, lack of chromosomally normal embryos (*n*=535, 1.9%) or abnormal endometrium (*n*=345, 1.2%). Therefore, out of 27,304 FET cycles, the overall CPR and delivery rate per transfer were 40.7% and 30.0%, respectively (Table 6), which is significantly higher than in 2018 (39.5% and 28.3%, respectively; both *p*<0.0001) and also significantly higher than the CPR (33.6%) and delivery rate (24.4%) after fresh transfers (both *p*<0.0001). The higher CPR and delivery rate for FET compared with fresh transfers were observed across all numbers of embryos transferred (Supplementary Table 4 and Table 3). The higher CPR and delivery rate in FET over fresh transfers was especially evident for SET. Furthermore, out of 8196 deliveries after FET reported in this period, 86.5% were singletons, 13.2% were twins and 0.3% were triplets and higher multiples.

### Outcome of freeze-all cycles

A total of 23,355 cycles of total embryo freezing were reported, 30.7% more than in 2018. On average 3.72 embryos (SD 2.83) were cryopreserved. During 2019, there were 6437 FET resulting from freeze-all cycles, giving rise to 2226 deliveries and a DR/ET of 34.6%, which is significantly higher than the DR/ET observed in non-freeze-all FET cycles (5970/20.867, 28.6%; *p*<0.00001). A second FET attempt with freeze-all embryos was reported in 1047 cycles from the same cohort, with 249 subsequent deliveries; the DR/ET in this attempt was 23.8%. Therefore, adding all the transfers from this subset of freeze-all cycles, the delivery rate per embryo transfer reaches 38.4% in women whose mean age was 39.9±4.85 years.

### Influence of stage of embryo development at transfer

Overall, 69.2% of embryo transfers were carried out at the blastocyst stage, representing a 17% increment over 2018. The proportion of blastocyst transfers for FET (80.8%) was almost double the proportion for fresh IVF/ICSI (46.3%). This is important to consider when comparing outcomes after the transfer of fresh embryos over FET. In oocyte donation cycles (including the transfer of fresh and frozen-thawed embryos), the proportion of blastocyst transfers reached 73.8%.

In autologous fresh IVF/ICSI, the delivery rate after 7963 blastocyst transfers was 29.6%, compared with 19.9% after the transfer of 9223 cleaving-stage embryos (*n*=9199) and zygotes (*n*=24; *p*<0.0001). For oocyte donation, the delivery rate per embryo transfer was 36.3% in blastocyst transfers and 23.9% in cleaving embryo transfers (*p*<0.0001); for FET cycles with autologous embryos, the rates were 32.6% and 20.1%, respectively (*p*<0.0001). For fresh cycles, the delivery rate was higher for blastocyst transfers alone compared with transfers at all embryo stages, irrespective of the number of embryos transferred (Tables 4 and 5). In all cases blastocyst transfer improved delivery rate.

### Influence of PGT on ART outcome

A total of 143 centres reported aspirations leading to PGT, in 14,135 out of 97,220 cycles (14.5%). From these procedures, there were 4073 embryo transfer cycles, including 198 fresh transfers and 3875 FET. Of these, 3423 transfers were from autologous cycles and 650 from oocyte donation. The mean age of women undergoing autologous PGT was 37.59 (SD 4.67) years, whereas for oocyte donation cycles with PGT the mean age of the donors was 25.7 (SD 3.67) years. In autologous cycles, a mean of 3.05 (SD 2.45) embryos were biopsied and the mean number of normal embryos was 1.81 (SD 1.29). In oocyte donations, a mean of 3.71 (SD 2.75) embryos were biopsied and the mean number of normal embryos increased to 2.44 (SD 1.75). The DR/ET was 33.5% and 35.4% in autologous and oocyte donation cycles, respectively.

The miscarriage rate using PGT was 10.7% after FET and 12.1% in oocyte donation FET. The effect of PGT on the rate of miscarriage after FET/oocyte donation as well as in different age groups for autologous cycles is presented in Table 7. When comparing miscarriage rates after autologous FET with and without PGT, the use of PGT resulted in significantly lower rates in women aged ≥35 years (both *p*<0.0001 for the 35-39 years and >39 years age groups). In women aged <35 years, the effect of PGT was of borderline significance (*p*=0.0513). Furthermore, there were 38 miscarriages in 313 pregnancies resulting from PGT carried out in oocyte donation FET (12.1%) compared with a miscarriage rate of 16.9% in FET oocyte donation without PGT (*p*=0.0351; 95% CI 0.5-8.5%).

**Table 7:** Effect of PGT on delivery rate and miscarriage rate after autologous FET and ODfrozen FET in different age groups.

Outcome	Age of women	FET with PGT %	FET without PGT %	*P*-value (95% CI)
Miscarriage	Oocyte donors	12.1 (38/313)	16.9 (555/3278)	0.0351 (0.5-8.5%)
	Autologous <35 years	11.2 (35/313)	15.5 (533/3431)	0.0513 (0.09-7.8%)
	Autologous 35-39 years	11.5 (72/624)	18.2 (773/4244)	<0.0001 (3.7-9.4%)
	Autologous >39 years	9.4 (45/478)	23.0 (462/2010)	<0.0001 (10.1-16.7%)
Delivery	Oocyte donors	37.7 (237/629)	31.6 (2457/7774)	0.0019 (2.2-10.2%)
	Autologous <35 years	33.4 (245/734)	34.1 (2592/7600)	0.7328 (-3% to 4.3%)
	Autologous 35-39 years	35.1 (498/1417)	30.4 (3122/10268)	0.0004 (2.1-7.4%)
	Autologous >39 years	33.2 (363/1095)	22.2 (1376/6190)	<0.0001 (8.0-14.1%)

For miscarriage rate the denominators are clinical pregnancies; for delivery rate, the denominators are embryo transfers.

FET, frozen embryo transfer; OD, oocyte donation (frozen); PGT, preimplantation genetic testing.

### Fertility preservation

A total of 7990 initiated cycles for fertility preservation were reported in 2019, representing a 19.5% increase over 2018. Of these, only 7531 cycles had one or more vitrified oocytes (459 cancelled cycles). The mean age of women was 36.1 years (≤34 years, 25.5%; 35-39 years, 50.9%; and ≥40 years 23.6%). No oocytes were available for cryopreservation for 471 follicular aspirations (5.9%). The mean number of oocytes cryopreserved was 7.7, with great variations depending on the age of the woman (≤34 years, 10.7 oocytes; 35-39 years, 7.5 oocytes; and ≥40 years, 4.8 oocytes).

**Figure 7:**
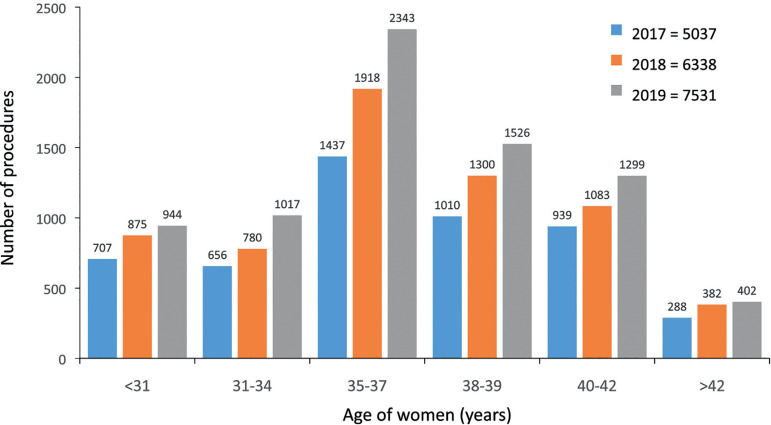
Age of women at fertility preservation in Latin America, 2017-2019.

In cases in which the indication for fertility preservation was recorded, most were related to the desire/need to postpone pregnancy (4766 cases, 63.4%), whereas cancer-related factors were reported in 428 (5.7%) cases, risk of premature ovarian insufficiency in 453 (6.0%) cases, and other reasons in 1872 cases (24.9%). More than 10 oocytes were cryopreserved in only 24.1% of women expressing the desire to postpone fertility and in 32.9% in women having cancer; as expected, the proportion dropped to only 8.4% in women with risks of premature ovarian insufficiency. Figure 7 includes all cycles between 2017 and 2019 where at least one oocyte was vitrified; 74% of these cycles were performed in women ≥35 years and 43% were in women aged ≥38 years.

### Cumulative live birth rate

The outcome of fresh embryo transfers and their consecutive FET was examined in a population of 59,105 women, followed between 2017 and 2019. This cohort included all women having fresh transfers irrespective of whether they had surplus frozen embryos resulting from their fresh transfer. The women were followed up to their first delivery after their fresh or frozen transfers. There were 15,973 births (92.5%) after fresh transfers and only 1293 births after FET in that same cohort. Taking all participants together, the birth per embryo transfer increased from 27.0% after a fresh embryo transfer to a cumulative rate of 29.2% (Relative Risk 3.6087; 95% CI 3.4188-3.8093; *p*<0.0001).

**Figure 8:**
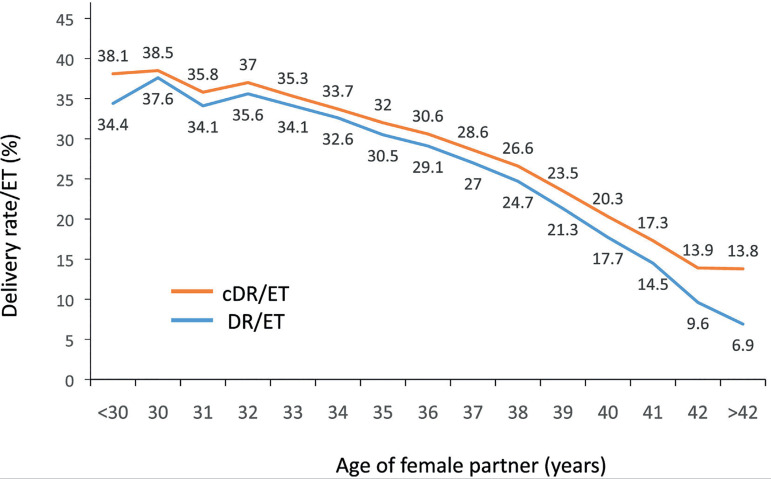
Delivery rate (DR/ET) and cumulative delivery rate (cDR/ET) per fresh embryo transfer in IVF/intracytoplasmic sperm injection cycles according to the age of the female partner in Latin America, 2017-2019.

The cumulative DR/ET stratified according to the age of the female partner at the time of oocyte retrieval is shown in Figure 8. The increment in delivery rate when adding FET to fresh transfers was in the order of 1-3%. In this cohort, there were 6014 women undergoing FET, of whom 5005 women (83.2%) had only one FET; 791 women (13.2%) had two FET and 218 women (3.6%) had three or more FET. The odds ratio showed a likelihood of delivery that was 1.3 times higher in women <35 years (95% CI 1.2-1.3), 1.2 in women aged 35-39 years (95% CI 1.1-1.3) and 1.1 in women ≥39 years (95% CI 1.1-1.3) with the addition of FET.

### Perinatal outcome and complications

Perinatal mortality is presented in Table 8. Data were available from 18,133 births and 21,096 babies born. The perinatal mortality increased from 7.4‰ of births in 15,260 singletons, to 17.2‰ in 5566 twins and 62.9‰ in 270 triplets and higher multiples. With 41 more babies born than in 2018, multiparity increased perinatal death in similar proportion to previous years.

**Table 8:** Perinatal mortality according to gestational order in 2019.

Outcome	Singleton	Twin	≥Triplet
Livebirth,^a^*n*	15,147	5470	253
Stillbirth, *n*	36	35	8
Early neonatal death, *n*	77	61	9
Perinatal Mortality^b^	7.4‰	17.2‰	62.9‰

a Early neonatal deaths are excluded.

b Perinatal mortality = (stillbirth + early neonatal death) / (livebirth + stillbirth + early neonatal death).

Gestational age at delivery was reported for 16,737 deliveries (92.3% of all deliveries; Table 9). The mean gestational age at delivery was 37.8 (SD 2.1) weeks for singletons, 35.4 (SD 2.8) weeks for twins, and 32.6 (SD 3.8) weeks for triplets and higher multiples. The overall risk of preterm birth (gestational weeks 22-36) increased from 14.3% for singletons to 58.1% for twins, and 81.1% for triplets and higher. Furthermore, the risk of very preterm birth (gestational weeks 22-27) increased from 0.7% for singletons to 2.1% for twins and to 7.8% for triplets and higher multiples.

**Table 9:** Gestational age and weight at birth according to gestational order in 2019.

Assisted reproductive technology procedure	Singleton		Twin		≥Triplets	
	Weeks of gestation, mean	Weight, mean ± SD (g)	Weeks of gestation, mean	Weight, mean ± SD (g)	Weeks of gestation, mean	Weight, mean ± SD (g)
Fresh autologous IVF/ICSI	37.7	3079 ± 580.6	35.4	2339 ± 516.6	31.3	1588 ± 551.6
Autologous FET	37.9	3207 ± 553.7	35.5	2365 ± 543.9	32.9	1463 ± 392.7
Fresh OD	37.4	3033 ± 582.0	35.2	2309 ± 557.5	33.0	1608 ± 366.2
Frozen OD	37.6	3048 ± 580.9	35.4	2260 ± 580.5	33.3	1633 ± 639.7
FTO	37.3	3061 ± 624.5	35.2	2234 ± 497.4	33.0	1687 ± 253.4
TOTAL	37.8	3128 ± 575.5	35.4	2326 ± 543.9	32.6	1570 ± 431.3

FET, frozen embryo transfer; FTO, includes embryo transfer cycles using vitrified-warmed oocytes; ICSI, intracytoplasmic sperm injection; OD, oocyte donation.

The weight of babies born from fresh and frozen-thawed embryos, from autologous reproduction and oocyte donation, as well as from FTO, is presented according to the order of gestation (Table 9). The weight of singletons born after FET (3207±554 g) was significantly higher than that of babies born after fresh transfer (3079±581 g; *p*<0.0001). Although the numbers of twins and triplets are lower, this difference was not seen for multiple births. Furthermore, the weight of singletons born after oocyte donation and FTO did not show differences in birthweight compared with fresh transfers in autologous reproduction.

## DISCUSSION

This is the 31^st^ report on ART procedures performed in Latin America. The number of new centres reporting to the RLA continues to grow. Between 2018 and 2019, six new centres were incorporated, contributing almost half of the new cycles reported in this period (2.6%). As seen in Figure 2, the number of initiated cycles reported by 15 countries represents approximately 85% of the estimated total number of cycles carried out in the region. This constitutes a noteworthy commitment of the centres, which have voluntarily reported year on year for more than 30 years.

The mean ART utilization in 12 countries where data are reliable (Figure 2) is only 221 initiated cycles per million population, which is well under the threshold of 1500 cycles per annum per million inhabitants proposed by the European Society for Human Reproduction and Embryology (ESHRE) to fulfil the needs of the population (The ESHRE Capri Workshop Group, 2001). This poor utilization results from a lack of affordability on the part of individuals deprived of state support.

In fact, Argentina and Uruguay, with laws providing universal coverage for fertility treatments, have increased their utilization rate to 490 and 558 cycles per million inhabitants, respectively. Chile, with only partial public coverage, is also increasing its utilization rate but at a slower pace, with only 372 cycles per million inhabitants. Indeed, a decision by the state recognizing the right to universal access to ART is not enough. The right to found a family must be harmonized with other sexual and reproductive rights, requiring an appropriate distribution of human resources and complex health facilities. That is the main reason why ART utilization in countries like Argentina and Uruguay is way below that of wealthier countries in Europe, Asia and Australia (Wyns *et al*., 2020).

Reporting on the efficacy of ART can be presented in many ways. Although there is overall agreement that a standardized outcome for ART is a healthy live birth, the main difficulty lies in what to use as a denominator and how to reach international agreement to compare these results from different latitudes. By incorporating Figure 1, this issue has been addressed. If the chosen denominator is an ‘initiated cycle’, the freeze-all cycles need to be removed because those women are not exposed to the chance of pregnancy, at least in that particular cycle. That accounts for 19,434 out of 47,241fresh IVF and ICSI cycles, which leaves us with 24,673 initiated cycles in which women had the real intention of becoming pregnant in that treatment cycle. If the freeze-all cycles are removed for oocyte donors, this gives a total of 15,267 exposed to the chance of pregnancy, representing 79.2% of the initiated cycles. All these clinical and biological variables need to be considered when counselling patients and comparing outcome results.

**Figure 9:**
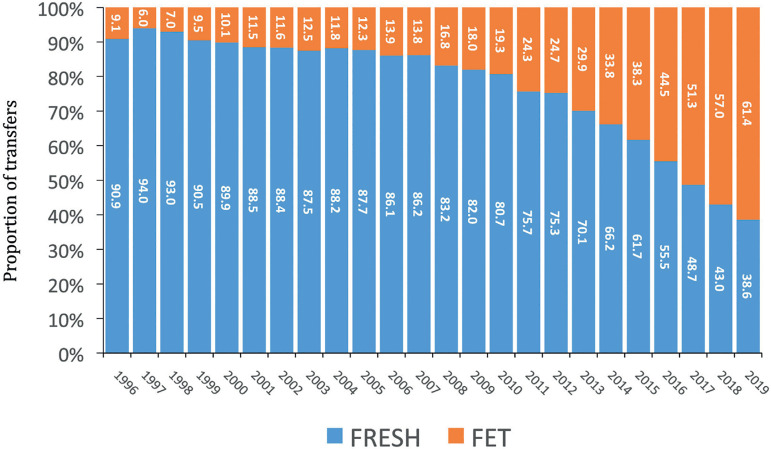
Proportion of fresh and frozen embryo transfer (FET) cycles in Latin America, 1996-2019.

When comparing fresh *versus* frozen thawed embryo transfer, Figure 9 shows that the proportion of FET over fresh transfers continues to rise, from 18% in 2009 to 61.4% in 2019. As shown in Table 2 compared with Supplementary Table 4, both CPR and DR/ET are significantly higher in the 27,304 FET compared with 23,819 fresh transfers. This is further confirmed when stratified by the number of embryos transferred. However, if fresh eSET is compared with FET SET, as seen in Table 4 and Supplementary Table 4, the delivery rate is higher in fresh eSET and even higher in blastocyst eSET (Table 5) than in FET SET; this suggests that it is always the quality of the embryos that matters most, irrespective of whether a transfer is fresh or frozen-thawed. This also provides further evidence that the cryopreservation technology does not affect embryo vitality.

In this reporting period, there were 23,355 freeze-all cycles, an approach that is being used as the first choice in many clinics today. In fact, there was a 30.7% increase over the previous year. In centres prioritizing fresh over FET transfers, the best embryos are transferred in the fresh attempt, while centres prioritizing FET transfers will freeze the best embryos for a delayed transfer. It is therefore understandable that the delivery rate in freeze-all cycles (34.6%) was significantly higher than in FET following a failed fresh cycle (28.6%). Similarly, the DR/ET in blastocyst transfers from freeze-all SET (1955/5092, 38.4%), was also significantly higher than in fresh blastocyst eSET (607/1858, 32.7%; *p*<0.0001; 95% CI 3.14-8.22%). These data strongly suggest that the chances of a birth are higher after the transfer of embryos that have been exposed to a freeze-all cycle compared with fresh transfers.

When comparing elective *versus* non-elective embryo transfer, as seen in Tables 4 and 5, the effectiveness of elective transfers is always greater than that of non-elective transfers, and the transfer of blastocysts is also beneficial. This is understandable because couples capable of generating numerous embryos are by definition more reproductively efficient than those generating only one good embryo, or two at the most. On the other hand, if embryo selection is performed at the blastocyst stage, the chances of birth are also higher. Furthermore, the transfer of one blastocyst is associated with a 1.8% chance of monozygotic twins compared with a 27.9% multiple birth rate when three selected blastocysts are transferred (Table 5). Therefore, when women can generate more than one blastocyst, eSET should prevail. The difference in birth rate for eSET compared with eDET is only eight percentage points, while the difference in multiple births rises from 1.8% to 27.9%, carrying all the accompanying perinatal mortality and morbidity derived from preterm births accompanied by increased health and social risks to the mother.

When examining cumulative live births in the cohort of 59,105 women followed between 2017 and 2019 (Figure 8), the vast majority of births took place after a fresh transfer (92.5%). The authors believe that the poor contribution of births after FET transfers results from a large proportion of older and/or reproductively inefficient women. In fact, of the 6014 women who underwent FET after the unsuccessful cycle, the vast majority (83.2%) had only one FET. When the cumulative birth rate was calculated in a subgroup of 20,906 women with at least one frozen embryo after their fresh transfer, the birth rate after fresh transfer in this cohort rose to 35% and the cumulative birth rate to 40.7%. Therefore, when comparing cumulative live birth rates, patient selection becomes a fundamental aspect to consider.

Concerning preimplantation genetic testing, a total of 143 reported 14,135 PGT cycles, representing an almost 60% increase over the previous year. There were 3875 FET cycles and, as seen in Table 7, the better outcome after PGT was highly significant in women aged ≥35 years, in terms of both increasing deliveries and lower miscarriage rates. This positive impact is not seen in women aged <35 years with autologous reproduction. To the authors’ surprise, there was a significant improvement in the delivery rate and miscarriage rate when PGT was performed in oocyte donation where the mean age of the donors was under 30 years. The authors’ clinical experience shows that young women are increasingly requesting PGT while performing ART procedures. The questions relate to how cost-efficient this is and what the role of reproductive health providers in advertising ‘certainty’ as an imperative value is.

The concept of fertility preservation deserves special attention. Data gathered from the last available 3 years (Figure 7) show that although fertility preservation increased by 50% between 2017 and 2019, the age of women requesting oocyte cryopreservation for non-medical reasons remained stable and very high. In Latin America, 74% of women freeze their oocytes at age ≥35 years and 43% at ≥38 years. Apart from the poor quality of oocytes at that age, the vast majority of women have only 5-8 vitrified oocytes. This implies that a large proportion of women are living with the unrealistic expectation of having a baby when they so wish. Public education and proper counselling from reproductive health professionals is very much needed.

In 2019, 65.4% of all multiple births took place in women <35 years of age as well as in oocyte recipients (data not shown in this manuscript). Therefore, it is in these women for whom blastocyst eSET should be implemented as the first option. Furthermore, the high birth rate after the transfer of frozen-thawed embryos in young women, which is similar to that in oocyte recipients, is reassuring. This indicates that blastocyst eSET or freeze-all eSET in these patients would result in acceptable cumulative birth rates and lower multiple births, thus generating a better balance between safety and efficacy. It is reassuring to realize that, year after year, the use of large and properly collected scientific data provides reliable evidence to offer safer and more efficient medical interventions.

## Appendix

**Supplementary Table 1:** List of centers reporting to the Latin American Registry (RLA)

ARGENTINA
• Servicio de Medicina Reproductiva, Instituto Gamma
• Centro de Estudios en Ginecología y Reproducción (CEGYR)
• Centro de Salud Reproductiva (CER)
• Instituto Tersoglio
• Centro Integral de Ginecología, Obstetricia y Reproducción (CIGOR)
• Centro de Investigaciones en Medicina Reproductiva (CIMER)
• Centro de Medicina Reproductiva Bariloche , Fertility Patagonia
• Centro de Estudios en Reproducción y Procedimientos de Fertilización Asistida (CRECER)
• FECUNDITAS
• FERTILAB
• GESTAR
• Centro de Reproducción Fertilequip
• Fertilis Medicina Reproductiva
• Fertya
• Hospital de Clinicas José de San Martin
• FECUNDART
• Centro de Reproducción, servicio de Ginecología Hospital Italiano
• Mater, Medicina Reproductiva
• Nascentis, Medicina Reproductiva
• HALITUS, Instituto Médico
• Instituto Medico de ginecología y Fertilidad PREFER
• PREGNA, Medicina Reproductiva
• Programa de asistencia reproductiva PROAR
• PROCREARTE
• Fertilidad San Isidro
• SARESA, Salud reproductiva Salta
• SEREMAS
• VITAE, Medicina Reproductiva
BOLIVIA
• CENALFES
• Instituto de Salud Reproductiva (ISARE)
• EMBRIOVID, centro integral de reproducción y especialidades médicas
BRASIL
• ANDROLAB, Clinica y Laboratorio de Reproducción Humana y Andrología
• ANDROFERT, Centro de Referencia en Reproducción Masculina
• FERTIVITRO, Centro de Reproducción Humana
• BIOS, Centro de Medicina Reproductiva
• FIV-MED
• Clinica Geare
• VIDA, Centro de Fertilidad
• Clinica FERTWAY
• ORIGINARE, Centro de Reproducción Humana
• CLINIFERT, Centro de Reproducción Humana
• CONCEPTUS, Centro de Reproducción Asistida de Ceara
• CONCEBER, Centro de Reproducción Humana
• Clinica Origen
• Clinica Pro-Genesis
• Centro de reproducción humana CONCEPTION
• Centro de Reproducción Humana MONTELEONE
• Fértile Diagnósticos
• CEERH, Centro especializado en Reproducción Humana
• Embrios, centro de reproducción humana
• EMBRYOLIFE, Instituto de Medicina Reproductiva
• CENAFERT, Centro de Medicina Reproductiva
• Instituto VERHUM
• Clinica FERTIBABY BH
• Fertilcare, Centro de reproducción humana Ltda.
• FECUNDA, Reproducción Humana
• FELICCITA, Instituto de Fertilidad Ltda.
• HUMANA, Medicina Reproductiva
• FERTILITY, Centro de Fertilización Asistida de Campo Grande
• FERTILITY, Centro de Fertilización Asistida
• FERTIL Reproduccion Humana
• REPROFERTY
• FERTICLIN, Clínica de Fertilidad Humana
• FECUNDAR Medicina Reproductiva
• GENESIS, Centro de Asistencia en Reproducción Humana
• Genics, medicina reproductiva y genómica
• FERTIPRAXIS
• GERA, Grupo de endoscopia y Reproducción Asistida
• Clinica GERAR VIDA
• Cegonha Medicina Reproductiva
• PRIMORDIA, Medicina Reproductiva
• Hospital de Clínicas de Riberao Preto
• HUNTINGTON Campinas
• HUNTINGTON, Centro de Medicina Reproductiva (Sao Paulo)
• JULES WHITE, Centro de Medicina Reproductiva
• HUNTINGTON Vila Mariana
• Ideia Fertil, Santo André
• Ideia Fertil, Sao Paulo
• IMR, Instituto de Medicina Reproductiva e Fetal
• Insemine , Centro de Reproducción Humana
• Centro de Reproducción Humana Santa Johana
• Life reproducción humana
• FERTILITAT, Centro de Medicina Reproductiva
• Clínica Nidus
• Centro de Reproducción Humana Nilo Frantz
• Origen, Centro de Medidicina Reproductiva BH
• Procriar, Centro de Medicina Reproductiva y diagnósticos Ltda., Blumenau
• Clínica PRO-CRIAR, Medicina Reproductiva BH
• Clínica PRO NASCER
• Clinica ProSer
• Centro de Reproducción Humana De San Jose de Rio Preto
• GENESIS, Centro de Reproducción Humana
• Centro de Reproducción Humana Prof. Franco Junior
• Centro de Ensino y Pesquisa en Reproducción Asistida (CEPRA)
CHILE
• UMR Clínica de la Mujer Antofagasta
• Centro de Estudios Reproductivos (CER)
• Unidad de Medicina Reproductiva, Clínica Alemana
• Unidad de Medicina Reproductiva, Clínica las Condes
• Unidad de Medicina Reproductiva, Clínica de la Mujer
• UMR clínica Indisa
• Programa e Fertilización Asistida I.D.I.M.I.
• Clínica Monteblanco
• Centro de Fertilidad y Medicina Reproductiva Concepción S.A.
• Centro de reproducción humana, Valparaiso
• SG Fertility Chile
COLOMBIA
• Centro FECUNDAR, Cali
• Unidad de fertilidad del Coutry ltda. CONCEPTUM
• FERTILITY CARE Colombia SAS
• Centro de fertilidad Clinica de la mujer
• Clinica Eugin
• Asociados en Fertilidad y Reproducción Humana
• FERTIVIDA
• Clinica Machicado SAS
• Centro Médico IMBANACO
• Instituto de Fertilidad Humana S.A.S. (INSER Bogotá)
• IN SER, Instituto Antioqueño de Reproducción (Medellín)
• Procrear
• Profamilia Fertil
• Unidad de Fertilidad, Procreación Medicamente Asistida
• Union temporal IN SER eje cafetero (Pereira)
ECUADOR
• Clínica de Medicina Reproductiva BIOGEPA
• Centro Ecuatoriano de reproducción humana
• Clínica INFES
• Instituto Nacional de Investigación de Fertilidad y Esterilidad (INNAIFEST)
• Instituto de Reproducción Humana Guayaquil
• CONCEBIR, Unidad de Fertilidad y Esterilidad
• Unidad de Fertilidad Hospital Alcívar
GUATEMALA
• Centro de Reproducción Humana S.A. (CER)
• Centro Clinico Gestar (nuevo)
MEXICO
• Centro de Diagnóstico Ginecológico
• Biofertility Center
• Clinica Cerh S e RL de CV
• URA, Unidad de reproducción asistida de Hispital CIMA Hermosillo
• Centro de Cirugía Reproductiva y Ginecología, Unidad de Fertilización In Vitro (REPROGYN)
• Instituto de Innovación Tecnológica y Medicina Reproductiva CITMER (Ciudad de México)
• Centro de Innovación tecnológica y medicina Reproductiva (Monterrey)
• Instituto para el estudio de la Concepción Humana IECH
• Centro de Reproducción Asistida del Hospital Español (HISPAREP)
• Centro de Reproducción Asistida del Occidente
• Centro de Reproducción Asistida de Saltillo
• Centro Universitario de Medicina Reproductiva
• Fertility Center Cancún
• Centro de Medicina reproductiva Filius
• Genesis Centro de Fertilidad (Culiacan)
• Ginecología y Reproducción Asistida GYRA
• Unidad de Medicina Reproductiva del Hospital Angeles del Pedregal
• IECH de Baja California
• Instituto Mexicano de Alta Tecnología Reproductiva S.C. (INMATER)
• Instituto de medicina reproductiva del Bajío IMER, sede Guadalajara
• Concibo
• Instituto Médico de la mujer (RED CREA)
• Iinstituto VIDA Guadalajara-Instituto de Ciencias en Reproducción Humana
• Instituto de Ciencias en Reproducción Humana, VIDA sede Matamoros
• Centro especializado para la atención de la mujer (CEPAM)
• INGENES DF
• INGENES Guadalajara
• Ingenes Monterrey
• Unidad de Reproducción Humana y Genetica, Poliplaza Medica (URHG)
• Instituto de Ciencias en Reproducción Humana (VIDA), sede León
• MasFertil
• Instituto de ciencias en reproducción humana del Sureste (Vida Merida)
• Clinica Nascere
• Plenus, Reproducción Asistida
• PROGEN, Reproducción asistida y medicina fetal
• Clinica de Infertilidad y reproducción asistida de Toluca SA de CV
• Centro especializado en esterilidad y Reproducción Humana (CEERH)
• Instituto de Ciencias en reproducción humana VIDA, ciudad de Mexico.
• Centro CARE
• Vida, Instituto de Reproducción Humana del Noroeste, Tijuana
NICARAGUA
• Centro de Fertilidad de Nicaragua
PANAMA
• IVI Panamá S.A.
• Centro de reproducción Punta Pacífica
• Instituto de salud femenina
• Centro Dr. Camilo Alleyne
PARAGUAY
• Neolife, Medicina y cirugía reproductiva
PERU
• Clínica CEFRA, Centro de Fertilidad y Reproducción Asistida
• CERFEGIN
• Centro de Fertilidad y Ginecología del Sur (CFGS)
• Clinica de fertilidad del norte, Clinifer de Chiclayo
• Centro de Fertilidad Germinar
• FERTILAB
• Inmater, Clinica de fertilidad
• Instituto de Reproducción de la Clinica Ricardo Palma
• Clinica Miraflores
• Nacer
• Grupo Pranor San Isidro, Clínica CONCEBIR
• Grupo Pranor, Instituto de Ginecología y Reproducción Monterrico
• Pranor, laboratorio de medicina reproductiva sede trujillo
REPUBLICA DOMINICANA
• Instituto de reproducción y ginecología del Cibao (IREGCI)
• PROFERT
URUGUAY
• Centro de Esterilidad Montevideo (CEM)
• Centro de Reproducción Humana del Interior
VENEZUELA
• FERTILAB
• Unidad de Fertilidad, UNIFERTES
• Instituto Venezolano de Fertilidad
• Laboratorio *In Vitro* de Venezuela

**Supplementary Table 2:** Clinical pregnancy rate (CPR), delivery rate and gestational order according to the number of embryos transferred in autologous frozen embryo transfer (FET) in 2019.

Number of embryos transferred	Embryo transfers		Clinical pregnancies		Deliveries							
	Number	%	Number	CPR (%)	Number of deliveries	Delivery rate per embryo transfer (%)	Singleton (n)	Singleton (%)	Twin (n)	Twin (%)	≥Triplets (n)	≥Triplets (%)
1	13,808	50.6	5250	38.0	3882	28.1	3819	98.4	62	1.6	1	0.02
2	12,192	44.7	5333	43.7	3947	32.4	2998	76.0	931	23.6	18	0.5
≥3	1304	4.7	517	39.6	367	28.1	273	74.4	89	24.2	5	1.4
Total	27,304	100	11,100	40.7	8196	30.0	7090	86.5	1082	13.2	24	0.3

**Supplementary Table 3:** Clinical pregnancy rate (CPR), delivery rate and gestational order according to the number of embryos transferred in fresh oocyte donations (OD) in 2019.

Number of embryos transferred	Embryo transfers		Clinical pregnancies		Deliveries							
	Number	%	Number	CPR (%)	Number of deliveries	Delivery rate per embryo transfer (%)	Singleton (n)	Singleton (%)	Twin (n)	Twin (%)	≥Triplets (n)	≥Triplets (%)
1	2525	40.1	1068	42.3	778	30.8	772	99.2	6	0.8	0	0
2	3187	50.6	1604	50.3	1194	37.5	811	67.9	376	32.5	4	0.3
≥3	583	9.3	285	48.9	222	38.0	123	55.4	80	36.0	9	4.1
Total	6295	100	2957	47.0	2194	34.9	1706	77.7	463	21.1	13	0.6

**Supplementary Table 4:** Clinical pregnancy rate (CPR), delivery rate and gestational order according to the number of frozen embryos transferred in oocyte donation (FET OD) in 2019.

Number of embryos transferred	Embryo transfers		Clinical pregnancies		Deliveries							
	Number	%	Number	CPR (%)	Number of deliveries	Delivery rate per embryo transfer (%)	Singleton (n)	Singleton (%)	Twin (n)	Twin (%)	≥Triplets (n)	≥Triplets (%)
1	4599	54.7	1804	39.2	1338	29.1	1324	99.0	13	1.0	1	0.1
2	3271	39.0	1524	46.6	1152	35.2	815	70.7	334	29.0	3	0.3
≥3	533	6.3	263	49.4	204	38.4	119	58.3	77	37.7	8	3.9
Total	8403	100	3591	42.7	2694	32.1	2258	83.8	424	15.7	12	0.4

## References

[r1] Dyer SJ, Chambers G, Zegers-Hochschild F, Adamson GD (2019). Access to ART: Concepts indicators, impact. Hum Reprod.

[r2] ESHRE Capri Workshop Group (2001). Social determinants of human reproduction. Hum Reprod.

[r3] Wyns C, Bergh C, Calhaz-Jorge C, De Geyter C, Kupka MS, Motrenko T, Rugescu I, Smeenk J, Tandler-Schneider A, Vidakovic S, Goossens V, European IVF-monitoring Consortium (EIM)‡ for the European
Society of Human Reproduction and Embryology (ESHRE) (2020). ART in Europe, 2016: results generated from European registries
by ESHRE. Hum Reprod Open.

[r4] Maheshwari A, McLernon D, Bhattacharya S (2015). Cumulative live birth rate: time for a consensus?. Hum Reprod.

[r5] Zegers-Hochschild F, Adamson GD, Dyer S, Racowsky C, de Mouzon J, Sokol R, Rienzi L, Sunde A, Schmidt L, Cooke ID, Simpson JL, van der Poel S (2017). The International Glossary on Infertility and Fertility Care,
2017. Hum Reprod.

[r6] Zegers-Hochschild F, Crosby JA, Musri C, Souza MDCB, Martinez AG, Silva AA, Mojarra JM, Masoli D, Posada N (2020). Assisted reproductive techniques in Latin America: The Latin
American Registry, 2017. JBRA Assist Reprod.

[r7] Zegers-Hochschild F, Crosby JA, Musri C, Souza MDCB, Martínez AG, Silva AA, Mojarra JM, Masoli D, Posada N (2021). Celebrating 30 years of ART in Latin America; and the 2018
report. JBRA Assist Reprod.

